# Reversible CT Scan Hypodensity in Acute Ischemic Stroke Patient With Low Initial Alberta Stroke Program Early CT Score (ASPECTS) Following Endovascular Thrombectomy: A Case Report

**DOI:** 10.7759/cureus.36194

**Published:** 2023-03-15

**Authors:** Mohammed S Alqahtani, Naif F Alharbi, Bashayer G Alghamdi, Muhannad A Asiri, Mohammed M Alwadai, Amani H Maghfuri, Saeed S Alzahrani

**Affiliations:** 1 Neurology, King Fahad General Hospital, Jeddah, SAU; 2 Neurology, Armed Forces Hospital-Southern Region, Khamis Mushait, SAU; 3 Radiology, King Saud Medical City, Riyadh, SAU

**Keywords:** mechanical thrombectomy, national institutes of health stroke scale (nihss), thrombolysis in cerebral infarction scale, stroke, tpa, tici score, endovascular thrombectomy, acute stroke, aspect score

## Abstract

According to current American and European guidelines, mechanical thrombectomy is recommended only for patients with an Alberta Stroke Program Early CT Score (ASPECTS) of 6 or higher. However, recent literature suggests that the potential benefits of reperfusion therapy should not be solely determined by baseline ASPECTS. In this case report, we present a young female patient with a low initial ASPECTS (4-5), who underwent mechanical thrombectomy and showed marked improvement in both CT imaging and clinical symptoms. Our findings potentially show that mechanical thrombectomy may be beneficial even for patients with an initial ASPECTS ≤ 5. These results may contribute to the growing evidence supporting the use of mechanical thrombectomy as a viable treatment option for acute ischemic stroke patients with low baseline ASPECTS.

## Introduction

Stroke is one of the leading causes of death and disability worldwide. From 1990 to 2019, the prevalence of stroke increased by 22% among individuals younger than 70 years [1], although ischemic stroke is commonly associated with elderly adults. Younger individuals between the ages of 18 and 50 are affected by stroke in an estimated 10-20% of cases., with cardioembolic source being the primary stroke mechanism [2,3]. The key characteristic of ischemic stroke diagnosis is a sudden onset of focal neurologic deficits [4], and the National Institutes of Health Stroke Scale (NIHSS) is a reliable, valid, and responsive tool for measuring stroke severity during emergency assessments [5].

Radiographic imaging is crucial in selecting appropriate thrombolytic and or endovascular treatments for ischemic stroke patients. The Alberta Stroke Program Early CT Score (ASPECTS) system is used to capture the extent of early ischemic changes based on visually evident hypoattenuation on CT scans. Low ASPECTS, indicating a large area of hypoattenuation, may make thrombolytic or endovascular therapy unsuitable due to increased treatment risks and limited benefits in terms of tissue preservation [6].

Improved outcomes have been observed in patients with large vessel occlusion (LVO) in the anterior circulation who undergo mechanical thrombectomy. However, based on current American and European guidelines, this treatment only applies to patients with ASPECTS of 6 or more [7]. The "fogging effect," in which a radiologically hypodense region on a CT scan of an infarct becomes an isodense region after two to three weeks, occurs in approximately 54% of all ischemic stroke patients [8]. Similarly, an immediate normalization of infarcted brain areas on CT scans after endovascular thrombectomy has been observed, which is associated with a better prognosis [9].

## Case presentation

**Figure 1:**
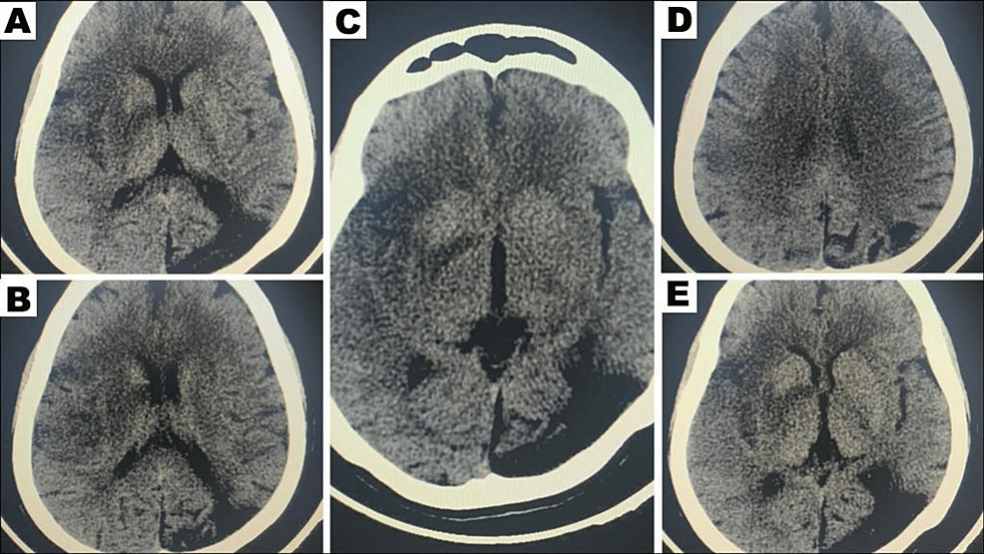
The initial CT head performed before mechanical thrombectomy revealed a low ASPECTS affecting the distribution of the right MCA. ASPECTS: Alberta Stroke Program Early CT Score; MCA: middle cerebral artery

A 39-year-old female patient with a past medical history of hypertension and a prior ischemic stroke without residual neurological deficit presented to the emergency department with an acute onset of left-sided face/arm/leg hemiplegia, and forced gaze deviation to the right, with a calculated NIHSS score of 18. The symptoms occurred 10 hours prior to the presentation. A non-contrast CT scan of the head demonstrated early ischemic changes in the right middle cerebral artery territory with no evidence of intracranial hemorrhage or mass effect. Her initial ASPECTS was 4-5 (Figure 1). The CT angiogram confirmed complete occlusion of the M1 segment of the right MCA, in line with the code stroke protocol.

**Figure 2:**
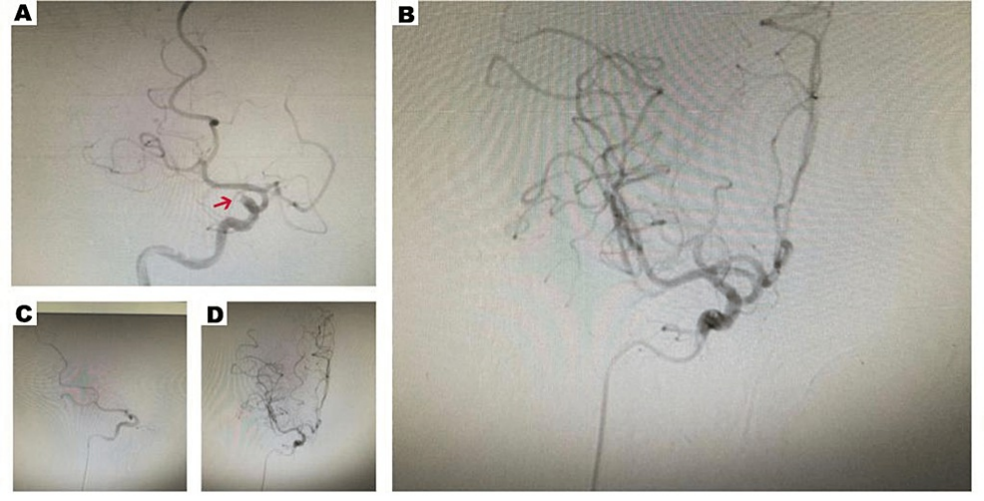
The right angiographic run images before (A,C) and after (B,D) successful mechanical thrombectomy of the right middle cerebral artery.

After a thorough discussion between the stroke neurologist, neurointerventionist, and the patient and her family, informed consent was obtained and mechanical thrombectomy was performed without administration of intravenous recombinant tissue plasminogen activator (IV tPA). Complete revascularization was achieved within approximately 15 minutes from the puncture, using a direct aspiration technique in one attempt. Angiography at the end of the intervention revealed a complete reperfusion of the occluded right middle cerebral artery (MCA) (modified thrombolysis in cerebral infarction (TICI) classification 2c) (Figure 2).

**Figure 3:**
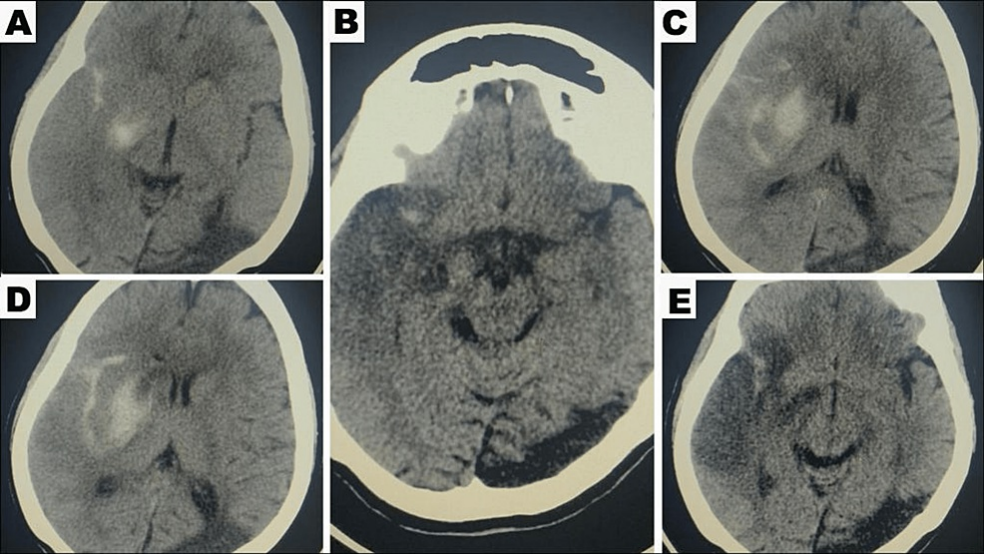
Initial CT head obtained 24 hours post procedure showed a mixed area of hypodensity with hyperdensity, mostly due to contrast leakage from her mechanical thrombectomy.

**Figure 4:**
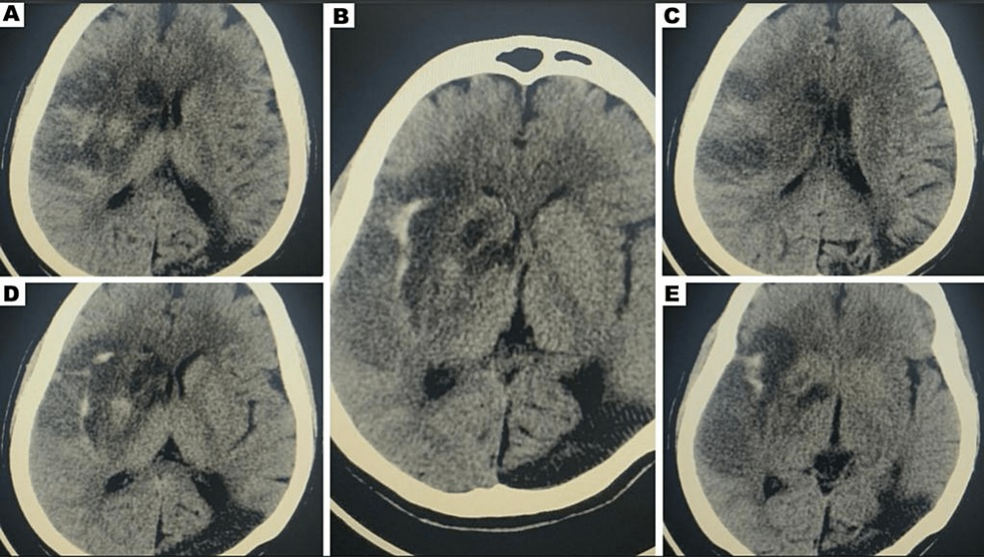
Two days post mechanical thrombectomy demonstrated well-established infarction over the right MCA territory. MCA: middle cerebral artery

**Figure 5:**
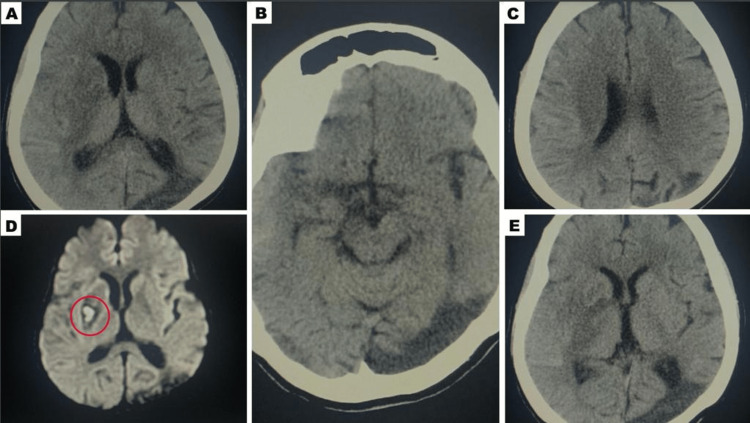
The CT head obtained on the sixth day showed almost complete reversal of the initial hypodensity observed in her scans (A,B); the CT head obtained on the eighth day demonstrated a similar pattern (C,E). Only a final small area of infarct was evident on her MRI brain (D), indicating that the initial hypodensity was reversible.

After a successful endovascular thrombectomy, an initial CT head was obtained 24 hours post procedure in accordance with post-thrombectomy protocol. The scan showed a mixed area of hypodensity with hyperdensity, mostly due to contrast leakage from her recent mechanical thrombectomy (Figure 3). Two days later, a well-established hypodensity was observed in the right MCA territory (Figure 4). Interestingly, repeated CT scans on the sixth and eighth days post procedure demonstrated a marked improvement in hypodensity, almost reaching isodensity, which was confirmed by MRI brain showing a small area of infarction, indicating the initial hypodensity was reversible (Figure 5).

Further comprehensive stroke workup revealed a cardioembolic source as the most likely stroke mechanism, based on echocardiography showing left ventricular ejection fraction of 55%, rheumatic heart disease with moderate to severe mitral stenosis, mild to moderate tricuspid regurgitation, mild mitral regurgitation, aortic regurgitation, and moderate pulmonary artery hypertension. Anticoagulation was started a week after stroke onset, and the patient was referred to a specialized cardiac center for further evaluation and management of her cardiac disease.

At discharge, the patient had marked improvement with a mild left facial asymmetry and mild drift in the left upper and lower limbs, resulting in a discharge NIHSS score of 3. Follow-up in the secondary stroke prevention clinic after two months showed almost complete recovery, with NIHSS and Modified Rankin Scale (mRs) scores of 1 and 0, respectively.

## Discussion

The patient in our case had a low initial ASPECTS (4-5) and high NIHSS score, but still demonstrated a reversal of the initial CT hypodensity and marked clinical improvement after undergoing mechanical thrombectomy. This finding is consistent with previous literature that suggests the benefit of mechanical thrombectomy in this group of patients

Recent studies have challenged the conventional guidelines that recommend mechanical thrombectomy only for patients with a baseline ASPECTS of 6 or more. The MR CLEAR (Multicenter Randomized Clinical Trial of Endovascular Treatment for Acute Ischemic Stroke in The Netherlands) registry, a retrospective observational cohort study conducted in 2022, concluded that reperfusion benefit was not determined by baseline ASPECTS [7]. Furthermore, a retrospective cohort study using data from the Stroke Thrombectomy and Aneurysm Registry (STAR) published in 2021 found that nearly 20% of patients with an ASPECTS of 2 to 5 achieved functional independence at 90 days with mechanical thrombectomy, particularly in patients with successful recanalization [10]. Another observational study published in 2019 found that successful recanalization among patients with ASPECTS ≤5 was associated with a significant reduction in edema formation and malignant infarctions, as well as improved clinical outcomes [11].

Studies based on diffusion-weighted imaging (DWI) ASPECTS have also shown promising results for mechanical thrombectomy in patients with low ASPECTS. One study found that patients with a low DWI ASPECTS who underwent mechanical thrombectomy had better outcomes compared to those treated medically [12]. Another similar study reported more favorable clinical outcomes among patients under the age of 70 [13]. Additionally, a study aimed at assessing the impact of mechanical thrombectomy in patients with initial DWI-ASPECTS ≤6 concluded that it is associated with a higher rate of favorable outcomes and a lower rate of mortality [14,15].

Moreover, some studies have challenged these guidelines. For instance, one published in 2021 suggested that water uptake measured by quantitative net water uptake on CT scan had higher diagnostic power than other parameters such as age, NIHSS, or ASPECTS [16]. Another study suggested that thrombectomy in patients with low ASPECTS may be guided by collateral score status. It found that in stroke patients with ASPECTS ≤5 and good collateral scores, endovascular recanalization reduced edema and improved clinical outcomes [17].

Additionally, a study that investigated the prognosis of patients with low ASPECTS and a different baseline infarct volume found that low ASPECTS combined with baseline infarct volumes ≤70 cc had a favorable prognosis [18]. Therefore, while ASPECTS is an important factor to consider in the selection of patients for mechanical thrombectomy, other factors such as collateral score status and baseline infarct volume may also influence the clinical outcome.

## Conclusions

Although ASPECTS is an important factor in selecting patients for mechanical thrombectomy, our case suggests that this treatment may also benefit patients with low initial ASPECTS and high NIHSS score. Recent studies have challenged conventional guidelines and shown that reperfusion benefits may not be determined solely by baseline ASPECTS. Further large randomized clinical studies are necessary to identify additional factors that influence outcomes and refine criteria for selecting patients for mechanical thrombectomy. Treatment decisions should also consider individual patient characteristics and potential benefits and risks.
